# Transmission Blocking Immunity in the Malaria Non-Vector Mosquito *Anopheles quadriannulatus* Species A

**DOI:** 10.1371/journal.ppat.1000070

**Published:** 2008-05-23

**Authors:** Tibebu Habtewold, Michael Povelones, Andrew M. Blagborough, George K. Christophides

**Affiliations:** Immunology and Infection, Division of Cell and Molecular Biology, Faculty of Natural Sciences, Imperial College London, London, United Kingdom; Stanford University, United States of America

## Abstract

Despite being phylogenetically very close to *Anopheles gambiae*, the major mosquito vector of human malaria in Africa, *Anopheles quadriannulatus* is thought to be a non-vector. Understanding the difference between vector and non-vector mosquitoes can facilitate development of novel malaria control strategies. We demonstrate that *An. quadriannulatus* is largely resistant to infections by the human parasite *Plasmodium falciparum*, as well as by the rodent parasite *Plasmodium berghei*. By using genetics and reverse genetics, we show that resistance is controlled by quantitative heritable traits and manifested by lysis or melanization of ookinetes in the mosquito midgut, as well as by killing of parasites at subsequent stages of their development in the mosquito. Genes encoding two leucine-rich repeat proteins, LRIM1 and LRIM2, and the thioester-containing protein, TEP1, are identified as essential in these immune reactions. Their silencing completely abolishes *P. berghei* melanization and dramatically increases the number of oocysts, thus transforming *An. quadriannulatus* into a highly permissive parasite host. We hypothesize that the mosquito immune system is an important cause of natural refractoriness to malaria and that utilization of this innate capacity of mosquitoes could lead to new methods to control transmission of the disease.

## Author Summary

Malaria is a mosquito-borne infectious disease that threatens almost half of the human population and kills 1 to 3 million people every year. In sub-Saharan Africa, where the vast majority of deaths occur, the capacity of mosquitoes to transmit malaria varies greatly even between closely related species. We compared the ability of malaria parasites to develop in two very closely related mosquitoes, one vector and one non-vector, and found that non-vector mosquitoes kill parasites at various stages, predominantly when they invade the mosquito midgut. This is achieved by parasite clearance, possibly by lysis in the midgut cells and by melanization, both of which are reactions of the mosquito immune system. This phenotype depends on heritable and dominant traits that can be passed on to vector/non-vector mosquito hybrids. We examined whether specific components of the mosquito immune system affect the resistance of these mosquitoes to infection. By silencing the activity of three immunity genes, we transformed mosquitoes of the resistant species into highly susceptible. Our results suggest that the mosquito immune system may affect refractoriness to malaria in non-vector mosquitoes. This innate capacity of mosquitoes to kill malaria parasites could be utilized in future integrated efforts to control and ultimately eradicate the disease.

## Introduction

The *Anopheles gambiae* Giles complex comprises seven mosquito species and several incipient species [1]–[3]. Sibling species are closely related to each other, are morphologically indistinguishable, and can crossbreed in captivity; however, they vary greatly in their capacity to transmit human malaria [3]–[5]. *An. gambiae sensu stricto* (henceforth *An. gambiae*) and *Anopheles arabiensis* are highly efficient vectors in sub-Saharan Africa and surrounding islands. Other species of this complex are only locally important vectors: *Anopheles melas* in western Africa, *Anopheles merus* in eastern Africa, and *Anopheles bwambae* in Uganda. Finally, *An. quadriannulatus* Theobald species A and B, found in southern Africa and Ethiopia, respectively, are exceptional in that they are considered medically unimportant: human malaria parasites have never been detected in wild caught *An. quadriannulatus* females [4]. Both species display characteristics that are believed to have existed in ancestral forms of the complex, *i.e*. standard chromosomal arrangements, disjointed distribution and adaptation to temperate climates [1]. Furthermore, they have been considered strictly zoophilic although recent laboratory and field studies report equal feeding preference for human and cattle [6]–[8]. Although laboratory reared *An. quadriannulatus* species A can be infected with cultured *P. falciparum*, the infection prevalence is significantly lower than in *An. gambiae* and *Anopheles stephensi* [9].

*Plasmodium* undergoes a complex developmental lifecycle in the mosquito. As shown for the rodent malaria parasite *P. berghei*, a standard laboratory model system, the parasite suffers substantial losses during its passage through the mosquito. The greatest reduction in parasite numbers occurs at the ookinete-to-oocyst transition stage [10]. Ookinetes, an invasive parasitic form, are often eliminated by lysis (and clearance) or melanization in the mosquito midgut epithelium, which are controlled by reactions of the mosquito innate immune system [11]. However, the few parasites that survive to reach the oocyst stage, a sessile parasitic form developing on the basal side of the midgut epithelium, multiply and produce thousands of sporozoites. When the oocysts burst, sporozoites are released to the haemolymph, invade the salivary glands and, upon subsequent mosquito bites, infect human hosts. Here, we investigate the mechanisms of refractoriness to *Plasmodium* in the malaria non-vector mosquito *An. quadriannulatus*. We show that refractoriness is controlled by partially dominant genetic traits and is manifest by clearance and melanization of ookinetes in the mosquito midgut as well as by killing of other parasitic stages developing later in the mosquito. The mosquito immune system appears to play a fundamental role in these reactions: inactivation of genes known to contribute to parasite killing in the malaria vector *An. gambiae* renders *An. quadriannulatus* a highly efficient vector of the rodent parasite *P. berghei*. We speculate that the same resistance traits may be present in wild vector populations at lower frequencies, since genetic selection for refractoriness apparently generates *An. gambiae* lines with phenotypes that are similar to that of *An. quadriannulatus* [12],[13]; these phenotypes can be reversed after silencing specific immunity genes [14]. Our data suggest that resistance to malaria may be an ancestral state of mosquitoes and prompt us to hypothesize that co-evolution and co-adaptation between the parasite and its insect host have lead to less refractory populations (and species) and successful malaria transmission.

## Results/Discussion

### *Plasmodium* killing in *An. quadriannulatus*

**Figure 1:**
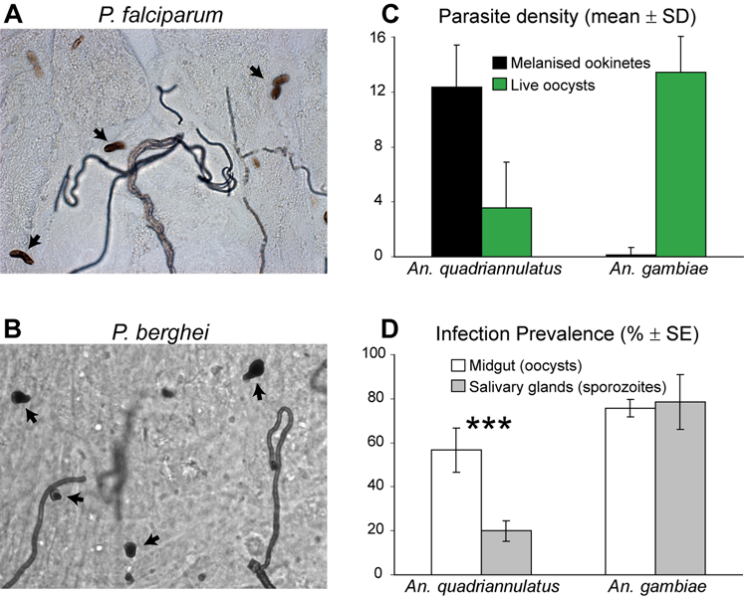
*Plasmodium* parasite killing in *An. quadriannulatus*. (A, B) Melanized ookinetes (arrows) of *P. falciparum* (A) and *P. berghei* (B) while crossing the *An. quadriannulatus* midgut. (C) Melanized ookinete and live oocyst density in the midguts of *An. quadriannulatus* and *An. gambiae* females infected with *P. berghei*. Four independent paired experiments were performed and their results were analysed by REML variance components analysis by fitting the mixed effect model. The geometric means±SD of the pooled data from the four independent experiments are shown. The melanized parasite densities (black bars) were 12.4±3.1 for *An. quadriannulatus* (n = 167) and 0.2±0.5 for *An. gambiae* (n = 118; P<0.001), and the oocyst densities were 3.6±3.3 and 12.8±2.6, respectively (P<0.001). n, number of midguts. (D) Prevalence (% of mosquitoes with at least one live parasite) of midguts at day 10 and salivary glands in corresponding infections at day 21–22 showing live *P. berghei* oocysts and sporozoites, respectively. The results of three independent experiments (see Table S1) were pooled and analyzed using the Chi-square goodness-of-fit test. A significant decrease in prevalence is detected in *An. quadriannulatus* (P<0.001), but not in *An. gambiae*. Bars represent standard errors.

We tested the ability of *An. quadriannulatus* species A, strain SKUQUA (henceforth *An. quadriannulatus*), to support development of *P. falciparum*. *An. gambiae* mosquitoes of the Yaoundé strain [15] were used as a reference. Three to four-day-old female mosquitoes were fed via a membrane with cultured *P. falciparum* gametocytes, and 10 days later their midguts were dissected and examined for oocysts. The results from two independent feeding experiments showed 0 of 18 (0/18) and 6/39 *An. quadriannulatus* midguts infected (0% and 15.4% infection prevalence, respectively); the corresponding median oocyst densities were 0.0 in both experiments (Table 1). In the paired feedings of *An. gambiae*, a known host for *P. falciparum*, 13/38 and 13/30 midguts had at least one viable oocyst (34.2% and 43.3% infection prevalence, respectively) with corresponding median oocyst densities of 5.0 and 12.0. Two subsequent *An. quadriannulatus* infections showed no live oocysts, but melanized ookinetes were occasionally observed in the mosquito midguts (Figure 1A); however, control *An. gambiae* mosquitoes were not used in these experiments, and thus comparisons cannot be made. Clearance of pre-oocyst parasitic stages and melanization of ookinetes are established important immune reactions of mosquitoes against *Plasmodium*. Thus, these data suggested that mosquito immunity could contribute to the reduced susceptibility of *An. quadriannulatus*.

**Table 1:** *P. falciparum* infection of *An. quadriannulatus* and *An. gambiae*.

Experiment	Species	n	Oocyst prevalence	P	Oocyst density	Range	P
1	*An. quadriannulatus*	18	0.0	ns	0.0	0	<0.01
	*An. gambiae*	38	34.2		5.0	1–43	
2	*An. quadriannulatus*	39	15.4	<0.001	0.0	1	<0.01
	*An. gambiae*	30	43.3		12.0		

Mosquito midguts were examined for *P. falciparum* live oocysts 10 days post-infection. Two independent experiments were performed. Oocyst prevalence is the percentage of mosquitoes displaying at least one live oocyst, and oocyst density is the median number of oocysts in infected mosquitoes. The range of oocyst numbers is also presented. In both experiments, the oocyst prevalence and density were substantially less in *An. quadriannulatus* compared to *An. gambiae*. Infection of *An. quadriannulatus* was not observed in experiment 1. The oocyst prevalence was analyzed with the Chi-square Fishers exact test with Yates correction, and the oocyst density with the Kruskal-Wallis non-parametric ANOVA. n, number of mosquitoes; ns not significant.

To investigate further this possibility, we utilized the convenient laboratory parasite, *P. berghei*, against which an extensive repertoire of mosquito immune responses has been previously documented. In these experiments, *An. quadriannulatus* and control *An. gambiae* mosquitoes were infected with a transgenic *P. berghei* parasite line that constitutively expresses green fluorescent protein (GFP) throughout its lifecycle [16]. Data from four independent infection experiments showed that *An. quadriannulatus* mosquitoes are highly refractory to *P. berghei*, in terms of oocyst prevalence, parasite density and ookinete melanization. A representative picture of an infected *An. quadriannulatus* midgut, with melanized *P. berghei* ookinetes, is shown in Figure 1B. The four experiments were analyzed by the Residual Maximum Likelihood (REML) variance components analysis, which revealed that the outcomes of these experiments were homogeneous and unlikely to be the result of random effects; thus justifying pooling of the data. Compared to *An. gambiae* (n = 118, where n is the number of midguts in the pooled data), *An. quadriannulatus* (n = 167) exhibited both reduced oocyst prevalence (67% vs. 100%; P<0.001) and increased ookinete melanization prevalence (93% vs. 13%; P<0.001) in their midguts 7–10 days post infection. As shown in Figure 1C, the density of melanized parasites per midgut of *An. quadriannulatus* was markedly greater than in *An. gambiae* (P<0.001) where melanization was only sporadically observed. In contrast, the live oocyst density was much lower (P<0.001) in *An. quadriannulatus* than in *An. gambiae*. The distributions of oocyst densities varied significantly (P<0.01) between the two mosquito species, as one-third of *An. quadriannulatus* had no oocysts whereas almost every *An. gambiae* midgut had one or more (Figure S1).

We assessed mosquito salivary gland infection by sporozoites to determine whether the losses of midgut parasitic stages in *An. quadriannulatus* can ultimately affect the transmission capacity of these mosquitoes. Importantly, we observed that parasite losses continue at later stages of the parasite lifecycle. In pooled data from three infection experiments (different from the above), the prevalence of *P. berghei* salivary gland sporozoites at day 21–22 post infection was much lower (20%; n = 115) compared to the prevalence of oocysts (57%; n = 104) at day 10 in the corresponding infection (P<0.001; Figure 1D and Table S1). No significant difference in prevalence between midgut (n = 105) oocysts and salivary gland (n = 85) sporozoites was detected in the paired infections of *An. gambiae* (76% vs. 79%, respectively). Moreover, salivary gland sporozoites in *An. quadriannulatus* appeared to be less infective compared to those in *An. gambiae*. From four bite-back experiments, using equal numbers of *An. quadriannulatus* or *An. gambiae* females (ranging from 10 to 15), which were infected 21–22 days earlier with *P. berghei* and then allowed to feed on naïve TO mice (one per experiment per mosquito species), only one resulted in mouse infection. In contrast, all four *An. gambiae* control bite-back experiments were infectious.

### Resistance to *Plasmodium* is heritable and dominant

A great variability in the degree of refractoriness to *P. berghei* was observed between *An. quadriannulatus* individuals, indicating genetic polymorphism within the mosquito population. The majority of mosquitoes in the four infection experiments described above displayed an intermediate phenotype with high numbers of both live oocysts and melanized parasites; others were fully resistant exhibiting strong ookinete melanization and no live oocysts, and yet others were highly susceptible, displaying many oocysts and few or no melanized ookinetes. No correlation (R^2^ = 0.001) was detected between oocyst and melanized ookinete counts (Figure S2). This variability within the population suggested that these phenotypes are determined by quantitative genetic traits. Two independent crossing experiments were carried out to determine whether the refractory traits of *An. quadriannulatus* are heritable. In these experiments, F1 females were generated by mass mating of *An. quadriannulatus* males and *An. gambiae* females (the reciprocal cross is uninformative because it predominantly yields males [17]). The resulting F1 females were then backcrossed to *An. quadriannulatus* males to obtain backcrossed F2 females. First generation F1 and F2 progenies, and the parental *An. quadriannulatus* and *An. gambiae* populations were compared for their ability to support parasite development (see Materials and Methods). The data resulting from the two crossing and infection experiments were pooled and analyzed with Kruskal-Wallis non-parametric ANOVA.

**Figure 2:**
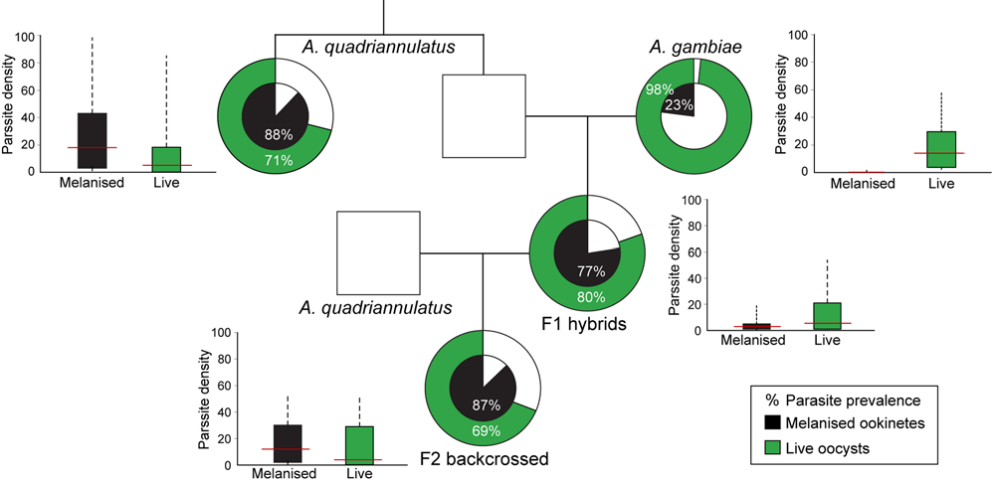
*An. quadriannulatus* refractoriness to *Plasmodium* is heritable and dominant. F1 hybrids were obtained by crossing male (square) *An. quadriannulatus* with female (circle) *An. gambiae*. F1 hybrid females were then backcrossed with *An. quadriannulatus* males to obtain F2 backcrossed mosquitoes. The prevalence of melanized parasites (inner pie, black) and live oocyst (outer pie, green) in the midguts of females from the parental *An. quadriannulatus* (n = 49) and *An. gambiae* (n = 53) populations, F1 (n = 66) and F2 (n = 86) are shown as concentric pie charts. Box plots depict the distribution of melanized ookinetes (black) and live oocysts (green) in each female mosquito group, with the respective median value shown in red. Two independent experiments were performed and the pooled data were analyzed using the Chi-square goodness-of-fit test for the prevalence and the Kruskal-Wallis non parametric ANOVA for the parasite densities. n refers to the number of mosquito midguts in the pooled data.

In terms of infection, the F1 hybrids were phenotypically similar to the *An. quadriannulatus* but different from the *An. gambiae* parental populations in both prevalence of infection (P<0.001) and oocyst density (P = 0.03; Figure 2). They displayed 80% oocyst prevalence with a median density of 5.5 per midgut (n = 66) compared to 71% and 5.0 in *An. quadriannulatus* (n = 49) and 98% and 14.0 in *An. gambiae* (n = 53), respectively. F1 and parental *An. quadriannulatus* mosquitoes exhibited marked similarity in their pattern of ookinete melanization. The prevalence of melanization was 77% in F1 hybrids and 80% in parental *An. quadriannulatus*; both of these were very different from the prevalence of melanization in *An. gambiae* (23%; P<0.001). A more striking similarity of refractoriness with the parental *An. quadriannulatus* was detected in the F2 backcrossed mosquitoes (n = 86; 69% oocyst prevalence and 4.0 oocyst density). Melanized ookinetes were detected in 75 of the 86 F2 females (87% prevalence) with median density 12.0 per midgut. All these measurements were significantly different from those reported above for *An. gambiae* (all at P<0.001 but the oocyst density which was at P<0.01). Together these data suggested that the refractoriness exhibited by *An. quadriannulatus* is heritable and that both traits contributing to this phenotype, reduction in the number of oocysts and increase in the number of melanized ookinetes, are dominant or partially dominant.

### Innate immunity controls resistance to *Plasmodium*

Given that the refractory mechanisms of *An. quadriannulatus* are under genetic control, we sought to determine if these are due to reactions of the mosquito innate immune system. Several *An. gambiae* genes have been implicated in lysis, clearance and melanization of *P. berghei* ookinetes in the mosquito midgut. In this initial study, we examined three genes, *LRIM1, LRIM2* (previously called *APL1* [13]; synonym is suggested here for systematization) and *TEP1*, all of which exhibit potent antagonistic effects against *P. berghei*. *LRIM1* and *LRIM2* encode leucine-rich repeat proteins, and silencing of either gene by RNA interference (RNAi) remarkably increases live oocyst densities in *An. gambiae* [13],[18]. *LRIM1* also mediates melanization of ookinetes in mosquitoes that are deficient for the melanization inhibitor C-type lectin 4, CTL4. TEP1 is the founder member of a thioester-containing protein family; it binds ookinetes promoting their lysis or melanization [14].

**Figure 3:**
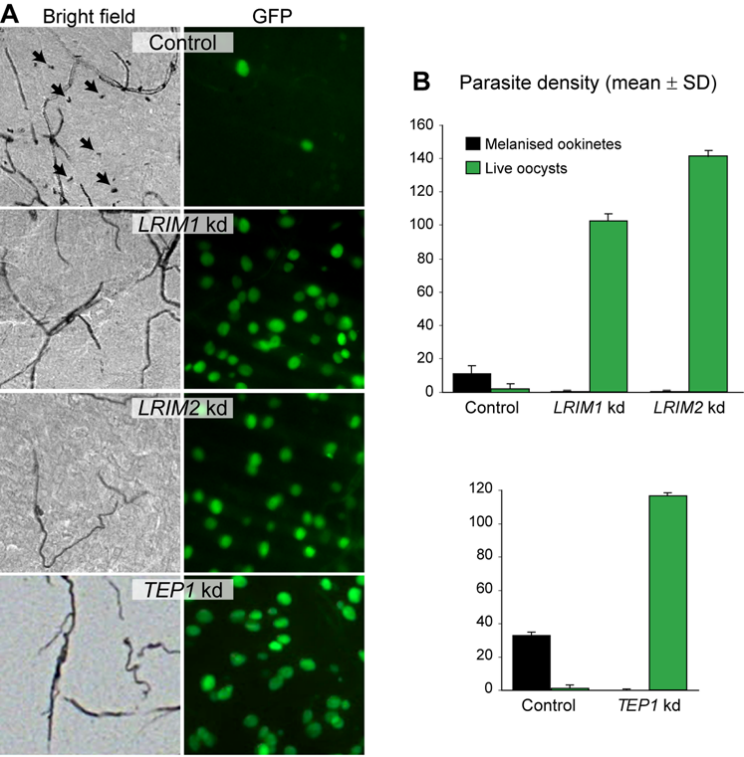
Silencing *LRIM1*, *LRIM2* and *TEP1* transforms *An. quadriannulatus* into a vector species. (A) Representative microscopy images of midguts dissected from *P. berghei*-infected mosquitoes which were injected with either *LacZ* dsRNA (control) or dsRNAs for each of the examined genes. GFP-fluorescent oocysts are shown in the right panels whereas arrows in the bright field images indicate melanized ookinetes. No melanized ookinetes and higher oocyst densities are observed in kd mosquitoes. (B) Quantitative effects of gene kds on melanized ookinete density (black bars) and oocyst density (green bars) in mosquito midguts compared to *LacZ* dsRNA-treated controls. The pooled results from two independent experiments were analyzed using the REML variance component analysis by fitting the mixed effects model. Geometric means and standard deviations are shown. Experiments with *LRIM1* and *LRIM2* kds were performed separately from those with *TEP1* kd; thus they are presented in separate graphs.

We used *An. gambiae*-specific oligonucleotide primers to amplify exon sequences of these genes from a cDNA pool constructed from *An. quadriannulatus* adult females. Sequencing the amplified fragments revealed a high degree of sequence similarity between *An. gambiae* and *An. quadriannulatus* for all the three genes: 98.9% for *LRIM1*, 96.2% for *LRIM2* and 98.7% for *TEP1* (Figure S3). This was not surprising as the two species are very closely related in the evolutionary scale and genetic introgression has likely taken place for some time after their separation. Using these gene fragments as templates, we produced double stranded RNA (dsRNA) sequences for each of these genes, which were microinjected separately in the body cavity of freshly emerged *An. quadriannulatus* females, as described for *An. gambiae* [19]. Mosquitoes injected with dsRNA of the *LacZ* gene were used as a control. Quantitative RT-PCR (qRT-PCR) revealed robust and specific silencing of cognate gene expression 4 days later, which ranged from 98% for *LRIM1* to 89% for *LRIM2*. As shown in Figure 3, silencing of *LRIM1*, *LRIM2* or *TEP1* in *An. quadriannulatus* resulted in a striking increase in *P. berghei* oocyst density (P<0.001) and complete inhibition of ookinete melanization compared to the control (P<0.001). While the oocyst density was 2.0 per midgut in control mosquitoes, this number increased to 102.5 in *LRIM1* and 141.0 in *LRIM2* knockdown (kd) mosquitoes. A similar increase was observed by silencing *TEP1* compared to the control: 116.5 vs. 1.1, respectively. These results suggest an effect for these genes in both ookinete melanization and clearance of parasites (possibly by lysis), as the number of oocysts in kd mosquitoes is much higher than the sum of oocysts and melanized ookinetes in the controls.

The very high sequence similarity between *An. gambiae* and *An. quadriannulatus*, which is expected for most genes in the two genomes, pointed to an intriguing possibility: that *An. gambiae*-specific dsRNA can be directly used to silence genes in *An. quadriannulatus*. Indeed, *An. gambiae*-specific dsRNA for *LRIM1* fully rescued the susceptibility phenotype when injected into *An. quadriannulatus* females, causing an approximately 4-fold increase in the oocyst density, 71% increase in oocyst prevalence (from 29% to 100%) and complete inhibition of ookinete melanization (data not shown). This result would constitute an important breakthrough if it pertains to additional genes: this non-vector species could then be utilized in conjunction with the *An. gambiae* vector as a model system, to further understand differences contributing to its reduced vectorial capacity.

Our results clearly indicate that a mosquito innate immune response accounts for most of the resistance of *An. quadriannulatus* to *P berghei*. *LRIM1*, *LRIM2* and *TEP1* are essential elements of this response and likely to operate in the same pathway, since their effects on the parasite are very similar. We examined with qRT-PCR whether the transcriptional profile of any of these three genes is different between the two mosquito species. In two independent experiments, mosquitoes were allowed to feed either on naive mice or on mice infected with *P. berghei*, and RNA samples from whole mosquitoes were prepared 24 hrs later. Sugar-fed mosquitoes of the same generation and age were also included. The results showed that the expression levels of *LRIM1* and *TEP1* were similar between the two species at all three conditions, with minor variations (Figure S4). However, the levels of *LRIM2* in sugar-fed and naive blood-fed mosquitoes were consistently elevated in *An. quadriannulatus* compared to *An. gambiae*. It remains to be explored whether this difference in *LRIM2* expression is directly related to the refractoriness phenotype of *An. quadriannulatus*. Furthermore, although only few nucleotide differences were identified in the sequenced gene fragments between *An. gambiae* and *An. quadriannulatus*, some of these differences in *LRIM1* and *LRIM2* lead to non-synonymous amino acid substitutions. Future research will aim to determine if any of these changes (or others in the non-sequenced gene segments) can enhance or otherwise alter the function of these genes, thus contributing to the refractoriness phenotype.

The *An. gambiae LRIM2* gene is located in a genomic region that was recently identified to control the density of mosquito infection with *P. falciparum* in a natural malaria transmission system in Mali, West Africa [13]. The same locus was responsible for almost 90% of parasite-free mosquitoes and 100% of mosquitoes with melanized parasites. These responses are highly similar to those we report here for *An. quadriannulatus*, making *LRIM2* a strong candidate for regulating natural mosquito refractoriness to the human malaria parasite. On the other hand, *LRIM1* was recently shown to have undergone strong positive selection in the other major African vector, *An. arabiensis*; this “arabiensis-like” allele has been introduced in *An. gambiae* populations at lower frequencies through multiple introgression events, but it is not present in other, less competent species of the complex including *An. quadriannulatus* [20]. These data in conjunction with the failure to demonstrate an apparent effect of *An. gambiae LRIM1* on sympatric field isolates of *P. falciparum* in a laboratory transmission setting in Cameroon [21], could suggest that *LRIM1* is subject to evolutionary adaptation to the human parasite; however, the effect of *LRIM1* on allopatric isolates or laboratory strains of *P. falciparum* is yet to be examined. Finally, *An. gambiae TEP1* was shown to have a strong antagonistic effect against a laboratory *P. falciparum* line [22]. Furthermore, a refractory allele of this gene is found in a genetically selected mosquito strain which kills and melanizes all *Plasmodium* species or strains that have been tested, except sympatric isolates of *P. falciparum* [12]. The phenotype of this refractory *An. gambiae* strain is identical to that of *An. quadriannulatus*. Therefore, it is tempting to speculate that persistent interaction of *An. gambiae* (and other major vectors) with *P. falciparum* might have led to an evolutionary co-adaptation between the mosquito immune responses and this parasite, whereas the resistance phenotype of the mostly zoophilic *An. quadriannulatus* could represent the ancestral function of the mosquito immune system against the parasite.

### Perspective

Malaria kills up to three million people every year and threatens the lives of almost half of the global population. Of the several hundreds of mosquito species only some anophelines can transmit human malaria. Even within the *An. gambiae* species complex, which includes some of the most important malaria vectors in Africa, the two *An. quadriannulatus* species are considered non-vectors. Researchers have proposed that understanding the differences between vector and non-vector mosquitoes could provide a new means for malaria control. Our research establishes for the first time a model laboratory system to study these differences at a genetic and molecular level. It demonstrates that mosquito immunity, which regulates the density of infection by the model rodent parasite, *P. berghei*, in the most competent vector of human malaria, *An. gambiae*, is the main cause of refractoriness to *P. berghei* in its non-vector sibling *An. quadriannulatus* species A. It remains to be revealed whether these findings also apply to infections with the human parasite *P. falciparum*.

## Materials and Methods

### Mosquito colonies, infections and dissections

The *An. quadriannulatus* SKUQUA strain was established from wild mosquitoes collected from an area near Skukuza, Kruger National Park, South Africa, in December 1995. The *An. gambiae* Yaoundé strain was colonized from wild mosquitoes collected from the Yaoundé area in 1988 [15]. Both mosquito colonies were raised at 28°C, 65–70% relative humidity, under a 12 hr light/dark cycle; adult mosquitoes were maintained on a 10% (w/v) sucrose solution. Infections with *P. berghei* were performed using the PbGFP*_CON_* parasite line [16], cultured using standard methods [23]. For infection, 50–70 female mosquitoes were randomly separated in paper caps, fed on anaesthetized mice infected with *P. berghei* (parasitaemia >5%) and kept at 20–21°C until the day of dissection. Midguts of mosquitoes were dissected 7–10 days post infectious blood meal, fixed in 4% para-formaldehyde and mounted on microscopy slides using VectaShield (Vector Laboratories Inc) before visualized with a light/fluorescence microscope. Killed parasite that appear as melanized ookinetes in the midguts and living oocyst that fluoresce green were separately quantified; melanized ookinetes were detected in bright field and oocysts were visualized with the fluorescein isothiocyanate filter.

For *P. falciparum* infections, erythrocytic stages of the 3D7 clone of the NF54 isolate were cultured as described [24], followed by induction of gametocytogenesis [25]. Cultures were then added to RBCs with HI AB serum at packed cell volume (ca. 40%) and introduced into membrane feeders. Mosquitoes were exposed to the membrane feeders for 25–30 min, and thereafter kept at standard insectary conditions until dissection. Mosquito midguts were dissected 7–9 days post infection, stained with 0.5% mercurochrome and examined for live oocysts and melanized ookinetes using a light microscope.

### Bite-back experiments

Detection of the infectivity of salivary gland sporozoites was carried out by mosquito bite-back experiments as described [26], with minor modifications. Female *An. quadriannulatus* and *An. gambiae* mosquitoes were infected with PbGFP*_CON_ P. berghei* after feeding on an infected TO mouse. Non blood-fed mosquitoes were removed. The presence of oocysts on the mosquito midguts was confirmed at day 8–10 post infection. At day 21–22 post infection, 10–15 of these mosquitoes were allowed to feed on naïve 8–10 week-old TO mice. The mice were then screened for blood staged parasites on day 5 after the mosquito bite, and the screening was continued every other day until day 15. The bite-back was considered non-infective if no blood-staged parasites were detected by day 15. Four independent replicate experiments were performed.

### Crossing experiments

In each crossing experiment, male *An. quadriannulatus* (300–400) and female *An. gambiae* (100–150) adult mosquitoes were allowed to mass mate to produce the F1 progeny. 100–150 females from this F1 progeny were then backcrossed to 300–400 *An. quadriannulatus* males to obtain the F2 progeny. In parallel to this backcrossing, the initial crossing of the parental populations was repeated in order to obtain first generation F1 progeny which were of the same age as the F2 progeny. Females of the parental populations and the F1 and F2 progenies were allowed to feed on *P. berghei*-infected mice as described above. Because there were four groups of females for each infection experiment, we used two mice of similar parasitaemia, each of which was randomly allocated to two of these groups; after 10 min in feeding, mice were swapped between group pairs. The entire crossing and infection experiment was repeated twice.

### DsRNA production and qRT-PCR

DsRNA production was performed as previously described, using gene specific oligonucleotide primers tailed with the short T7 promoter sequence TAATACGACTCACTATAGGG [27]. The sequences of these primers are: *LRIM1* F, AATATCTATCTCGCGAACAATAA; *LRIM1* R, TGGCACGGTACACTCTTCC; *LRIM2* F, GCTTACGCGCACACTATTCA; *LRIM2* R, GCTATTGTGCGATGCGTCTA; *TEP1* F, TTTGTGGGCCTTAAAGCGCTG; *TEP1* R, ACCACGTAACCGCTCGGTAAG; *LACZ* F, AGAATCCGACGGGTTGTTACT; *LACZ* R, CACCACGCTCATCGATAATTT. Injection of dsRNA in adult female mosquitoes was performed as described [19]. The aforementioned primers were also used to PCR amplify and determine the sequence of the *An. quadriannulatus* genes. Two additional primers were used for sequencing another fragment of the *LRIM1* gene in *An. quadriannulatus*, which was not part of the dsRNA-targeted sequence: *LRIM1* 988 F, ATCGCGCTGAAGCGCAAAGAG; *LRIM1* 1530 R, TTATCCCAGCTGGCTCGCTAAATTCTG.

qRT-PCR was performed as described previously [27], with the following modifications. Total RNA was extracted from approximately 10 adult mosquitoes with 1 ml of TRIzol reagent (Invitrogen) and treated with Turbo DNAfree (Ambion) according the manufacturer's directions. 1 µg of total RNA was used for reverse transcriptions using Superscript II (Invitrogen). Transcript abundance was measured with an Applied Biosystems 7700 Real-Time PCR system using the ribosomal S7 gene as an internal control. Reactions of 25 µl consisted of 1× SYBR green mix (Applied Biosystems) and cDNA, corresponding to 2.5 ng of total RNA. The primer sequences and concentrations in the final reaction are: *LRIM1* 1914 F (0.9 µM), CATCCGCGATTGGGATATGT; *LRIM1* 1983 R (0.9 µM), CTTCTTGAGCCGTGCATTTTC; *LRIM2* 825 F (0.9 µM), GCAAAGAAAGTGACAAGCCGTAT; *LRIM2* 884 R (0.3 µM), CGCTCGTCAGGGCAATGTA; *TEP1* 2676 F (0.9 µM), AAAGCTGTTGCGTCAGGG; *TEP1* 2750 R (0.3 µM), TTCTCCCACACACCAAACGAA; S7 F (0.3 µM), GTGCGCGAGTTGGAGAAGA; S7 R (0.3 µM): ATCGGTTTGGGCAGAATGC.

### Data analysis

For analysis of the data, the prevalence of infection and parasite density were treated as two independent infection variables, although they are likely to be partly connected. The prevalence data were analysed using the chi-square goodness-of-fit test, except for comparing the prevalence of *P. falciparum* infection between *An. quadriannulatus* and *An.* gambiae where Fisher exact test with Yates correction was used. For the analysis of density of oocysts and melanised ookinetes, mosquitoes showing no parasites (neither live oocysts nor melanised ookinetes) in their midguts were excluded, and the data were subjected to normality and homogeneity tests. As counts of both live and dead parasites (x) displayed right-skewed distributions, the geometric means were computed after data normalization by log_10_(x+1) transformation. The log-transformed data from all replicates within a study (dataset) were analyzed by REML (Rresidual Maximum Likelihood) variance components analysis by fitting the mixed effect model. In this model, we treated mosquito species or the control kd status as a fixed effect and introduced a random effect for the replicates. For each dataset a combined P-value is reported for the fixed effects. When normality of datasets could not be achieved by the above transformation method (i.e. for the *P. falciparum* oocyst density and for the density of oocysts and melanised ookinetes in the crossing experiments) the median value of untransformed data was computed and datasets were subjected to Kruskal-Wallis one-way ANOVA. Comparison of oocyst and melanised ookinete distributions between *An. quadriannulatus* and *An. gambiae* midguts was carried out using the Kolmogorov-Smirnov (KS) test. All these statistical analyses were performed using the GenStat® software.

## Supporting Information

Figure S1 Distributions of *P. berghei* oocysts in mosquito midguts. Midguts of *P. berghei*-infected *An. quadriannulatus* (n = 167) and *An. gambiae* (n = 118) were dissected 10 days post infection and oocysts were visualized. The midguts were grouped into successive bins according to their oocysts density. Kolmogorov-Smirnov (KS) statistical test reveals that the oocyst distributions are significantly different between the two mosquito species (P<0.01).
(3.85 MB TIF)

Figure S2 No correlation between melanized ookinete and oocyst densities. Corresponding densities of *P. berghei* live oocysts and melanized ookinetes in the midguts of *An. quadriannulatus* mosquitoes. The absence of correlation between these two phenotypic measurements suggests that they are genotypically unrelated.
(3.56 MB TIF)

Figure S3 *LRIM1*, *LRIM2* and *TEP1* are highly conserved between the two mosquitoes. Alignment of nucleotide and deduced aminoacid sequences of *LRIM1* (A, B), *LRIM2* (C) and *TEP1* (D) gene fragments between *An. gambiae* (*Ag*) and *An. quadriannulatus* (*Aq*). The gene fragments shown in A, C and D correspond to those used for dsRNA construction. Non-synonymous nucleotide differences are highlighted in red and synonymous differences are highlighted in grey.
(0.26 MB PDF)

Figure S4 Transcriptional profiles of *LRIM1*, *LRIM2* and *TEP1* in *An. gambiae* vs. *An. quadriannulatus*. Analysis of the relative transcription levels of *LRIM1* (A), *LRIM2* (B) and *TEP1* (B) in *An. gambiae* (white bars) and *An. quadriannulatus* (grey bars) female mosquitoes. The expression was assessed in sugar-fed mosquitoes, and mosquitoes fed 24 hrs earlier either on naïve or *P. berghei*-infected mice. Transcripts of the S7 ribosomal protein gene were used as internal normalization control. All data points for each gene were calibrated to the transcript levels in sugar-fed *An. gambiae*, which were set at 100%. The standard error of two independent experiments is shown.
(2.01 MB TIF)

Table S1 Prevalence of *An. quadriannulatus* and *An. gambiae* infection with *P. berghei*. Mosquito midguts were dissected 10 days post-infection and salivary glands were dissected 21–22 days post-infection to determine the prevalence of live oocysts and score the presence of sporozoites, respectively. Three independent experiments were performed. Prevalence values show the percentage of mosquitoes displaying *P. berghei* oocysts or salivary gland sporozoites, respectively; these values within each species were compared using the Chi-square goodness-of-fit test. n, number of midguts and salivary glands (SG); ns, not significant.
(0.06 MB DOC)
