# Supplementary figures and images for: Transmission Blocking Immunity in the Malaria Non-Vector Mosquito Anopheles quadriannulatus Species A
Source: PLoS Pathog. 2008 May 23;4(5):e1000070. doi: 10.1371/journal.ppat.1000070 (PMC2374904; doi:10.1371/journal.ppat.1000070)

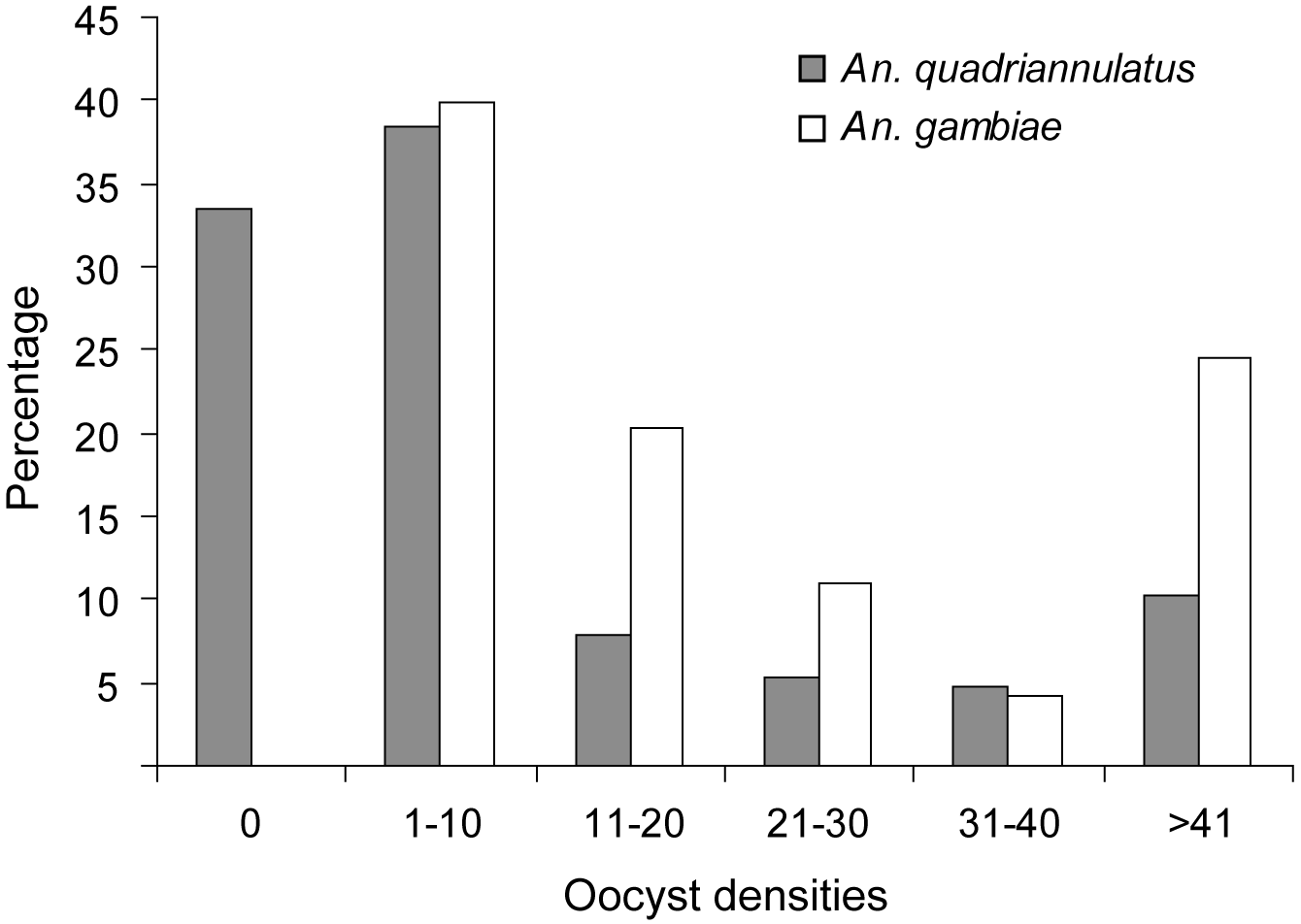

Supplement: Figure S1 — Distributions of P. berghei oocysts in mosquito midguts. Midguts of P. berghei-infected An. quadriannulatus (n = 167) and An. gambiae (n = 118) were dissected 10 days post infection and oocysts were visualized. The midguts were grouped into successive bins according to their oocysts density. Kolmogorov-Smirnov (KS) statistical test reveals that the oocyst distributions are significantly different between the two mosquito species (P<0.01). (3.85 MB TIF) [file ppat.1000070.s001.tif]

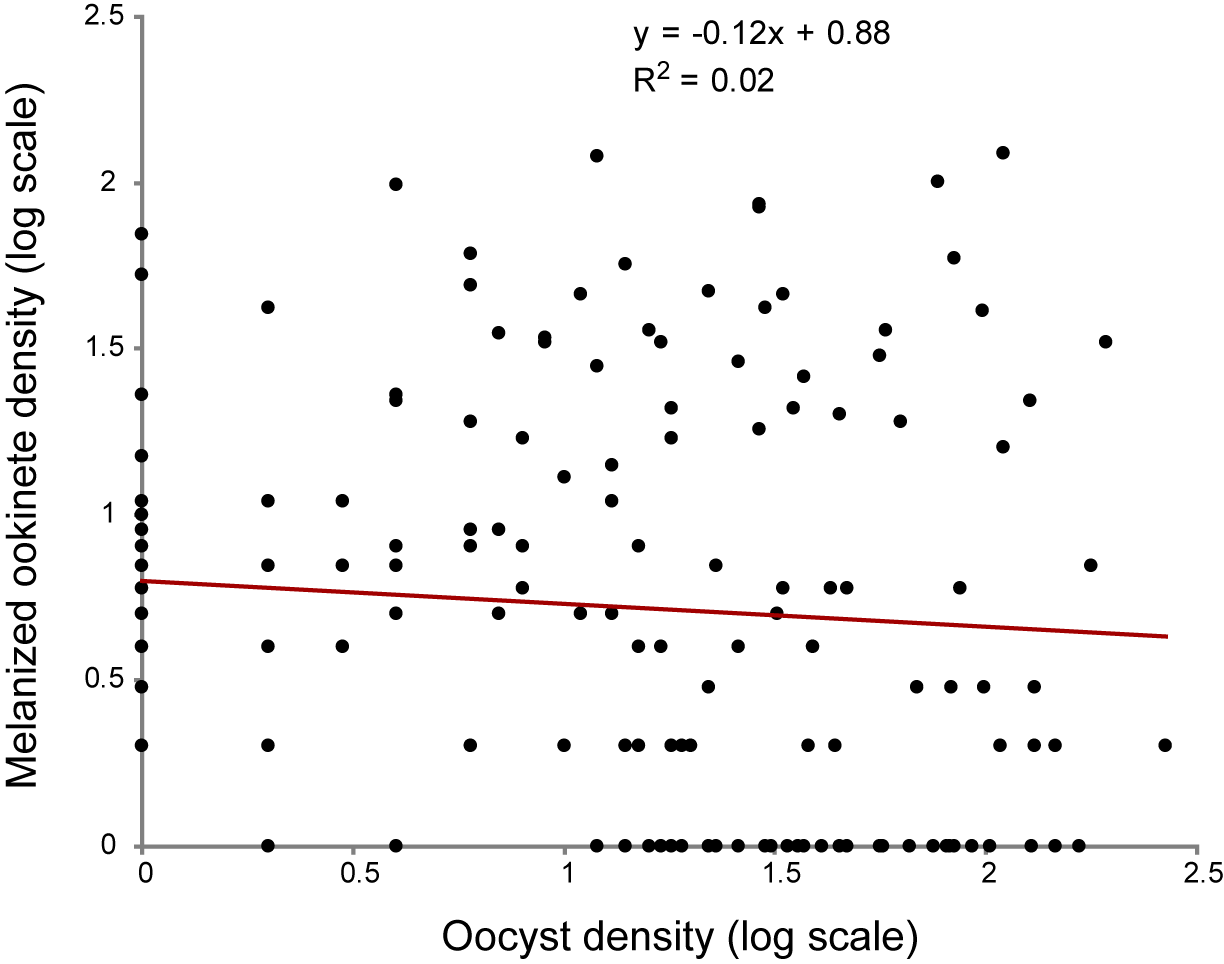

Supplement: Figure S2 — No correlation between melanized ookinete and oocyst densities. Corresponding densities of P. berghei live oocysts and melanized ookinetes in the midguts of An. quadriannulatus mosquitoes. The absence of correlation between these two phenotypic measurements suggests that they are genotypically unrelated. (3.56 MB TIF) [file ppat.1000070.s002.tif]

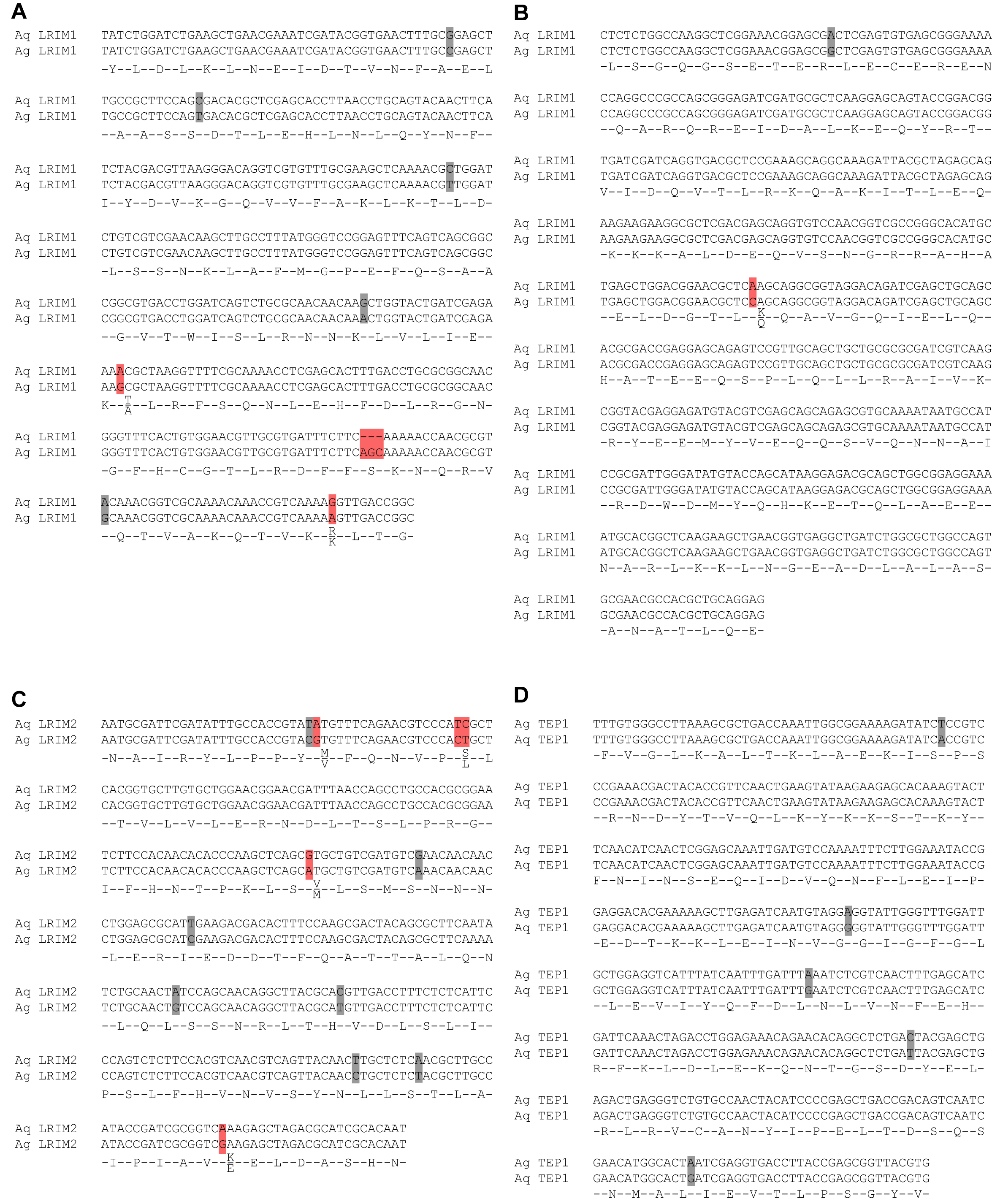

Supplement: Figure S3 — LRIM1, LRIM2 and TEP1 are highly conserved between the two mosquitoes. Alignment of nucleotide and deduced aminoacid sequences of LRIM1 (A, B), LRIM2 (C) and TEP1 (D) gene fragments between An. gambiae (Ag) and An. quadriannulatus (Aq). The gene fragments shown in A, C and D correspond to those used for dsRNA construction. Non-synonymous nucleotide differences are highlighted in red and synonymous differences are highlighted in grey. (0.26 MB PDF) [file ppat.1000070.s003.tif]

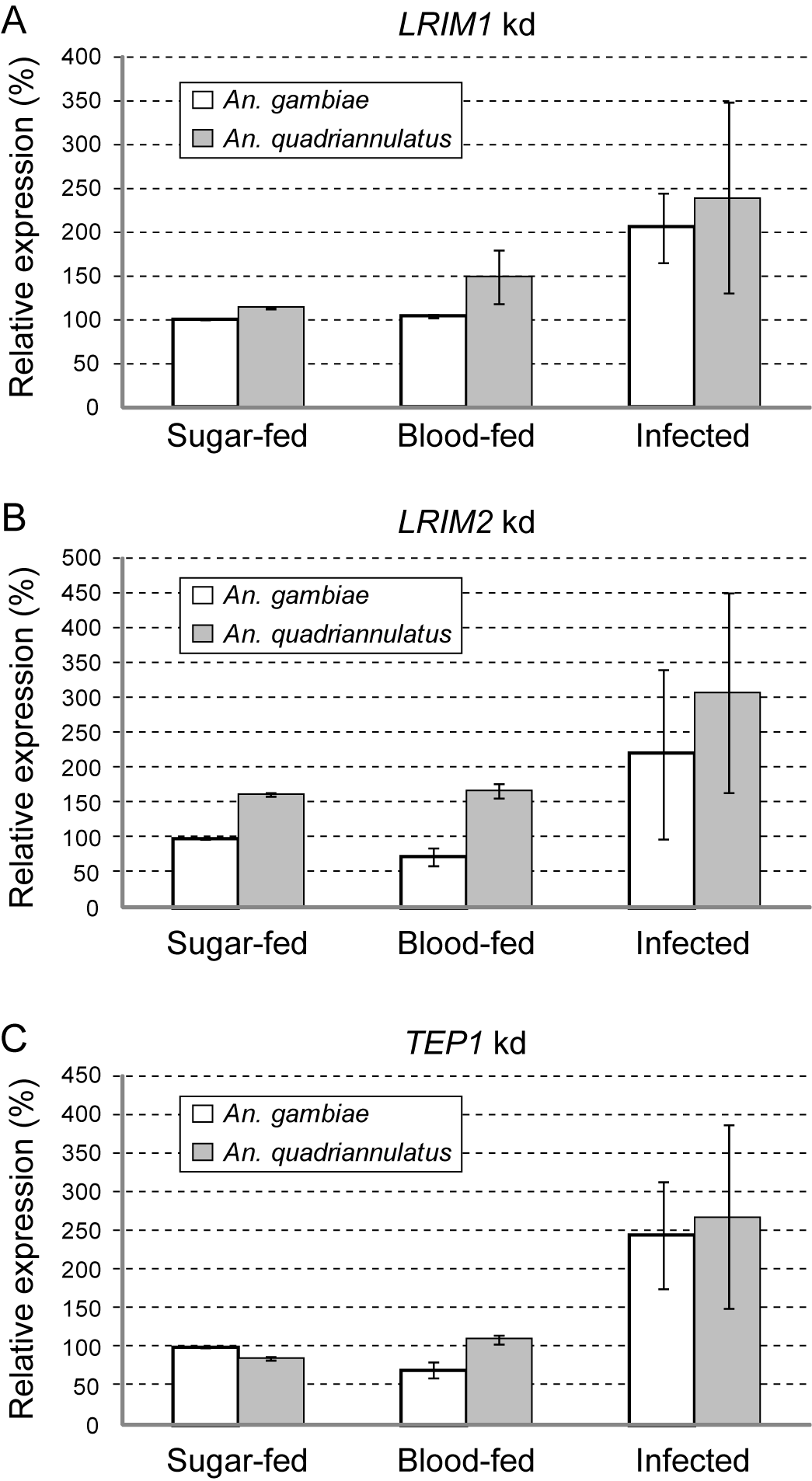

Supplement: Figure S4 — Transcriptional profiles of LRIM1, LRIM2 and TEP1 in An. gambiae vs. An. quadriannulatus. Analysis of the relative transcription levels of LRIM1 (A), LRIM2 (B) and TEP1 (B) in An. gambiae (white bars) and An. quadriannulatus (grey bars) female mosquitoes. The expression was assessed in sugar-fed mosquitoes, and mosquitoes fed 24 hrs earlier either on naïve or P. berghei-infected mice. Transcripts of the S7 ribosomal protein gene were used as internal normalization control. All data points for each gene were calibrated to the transcript levels in sugar-fed An. gambiae, which were set at 100%. The standard error of two independent experiments is shown. (2.01 MB TIF) [file ppat.1000070.s004.tif]
