# Supplementary material for: Transmission Blocking Immunity in the Malaria Non-Vector Mosquito Anopheles quadriannulatus Species A
Source: PLoS Pathog. 2008 May 23;4(5):e1000070. doi: 10.1371/journal.ppat.1000070 (PMC2374904; doi:10.1371/journal.ppat.1000070)
Supplement: Table S1 — Prevalence of An. quadriannulatus and An. gambiae infection with P. berghei. Mosquito midguts were dissected 10 days post-infection and salivary glands were dissected 21–22 days post-infection to determine the prevalence of live oocysts and score the presence of sporozoites, respectively. Three independent experiments were performed. Prevalence values show the percentage of mosquitoes displaying P. berghei oocysts or salivary gland sporozoites, respectively; these values within each species were compared using the Chi-square goodness-of-fit test. n, number of midguts and salivary glands (SG); ns, not significant. (0.06 MB DOC) [file ppat.1000070.s005.doc]

| **Table S1.** Prevalence of *An. quadriannulatus* and *An. gambiae* infection with *P. berghei* | | | | | | |
| --- | --- | --- | --- | --- | --- | --- |
|  |  | **Midgut** | **Oocyst** | **SG** | **Sporozoite** |  |
| **Experiment** | **Species** | **n** | **Prevalence** | **n** | **Prevalence** | **P** |
| 1 | *An. quadriannulatus* | 31 | 35.8 | 55 | 12.7 | <0.001 |
|  | *An. gambiae* | 31 | 80.7 | 50 | 86.0 | ns |
| 2 | *An. quadriannulatus* | 54 | 70.7 | 29 | 27.6 | <0.001 |
|  | *An. gambiae* | 52 | 69.2 | 20 | 45.0 | ns |
| 3 | *An. quadriannulatus* | 19 | 52.6 | 31 | 25.8 | ns |
|  | *An. gambiae* | 22 | 68.8 | 15 | 53.3 | ns |
| Pooled | *An. quadriannulatus* | 104 | 56.7 | 115 | 20.0 | <0.001 |
|  | *An. gambiae* | 105 | 75.7 | 85 | 78.5 | ns |
| Mosquito midguts were dissected 10 days post-infection and salivary glands were dissected 21-22 days post-infection to determine the prevalence of live oocysts and score the presence of sporozoites, respectively. Three independent experiments were performed. Prevalence values show the percentage of mosquitoes displaying *P. berghei* oocysts or salivary gland sporozoites, respectively; these values within each species were compared using the Chi-square goodness-of-fit test. n, number of midguts and salivary glands (SG); ns, not significant. | | | | | | |
